# Spatiotemporal variation in diabetes mortality in China: multilevel evidence from 2006 and 2012

**DOI:** 10.1186/s12889-015-1982-0

**Published:** 2015-07-10

**Authors:** Maigeng Zhou, Thomas Astell-Burt, Peng Yin, Xiaoqi Feng, Andrew Page, Yunning Liu, Jiangmei Liu, Yichong Li, Shiwei Liu, Limin Wang, Lijun Wang, Linhong Wang

**Affiliations:** National Center for Chronic and Noncommunicable Disease Control and Prevention, Chinese Center for Disease Control and Prevention, 27 Nanwei Road, Xicheng District, Beijing 100050 China; School of Science and Health, University of Western Sydney, Sydney, Australia; School of Geography and Geosciences, University of St Andrews, St Andrews, UK; School of Health and Society, University of Wollongong, Wollongong, Australia

**Keywords:** Diabetes, Mortality, Geographical variation, Trends, Risk factors

## Abstract

**Background:**

Despite previous studies reporting spatial in equality in diabetes prevalence across China, potential geographic variations in diabetes mortality have not been explored.

**Methods:**

Age and gender stratified annual diabetes mortality counts for 161 counties were extracted from the China Mortality Surveillance System and interrogated using multilevel negative binomial regression. Random slopes were used to investigate spatiotemporal variation and the proportion of variance explained was used to assess the relative importance of geographical region, urbanization, mean temperature, local diabetes prevalence, behavioral risk factors and relevant biomarkers.

**Results:**

Diabetes mortality tended to reduce between 2006 and 2012, though there appeared to be an increase in diabetes mortality in urban (age standardized rate (ASR) 2006–2012: 10.5–13.6) and rural (ASR 10.8–13.0) areas in the Southwest region. A Median Rate Ratio of 1.47, slope variance of 0.006 (SE 0.001) and covariance of 0.268 (SE 0.007) indicated spatiotemporal variation. Fully adjusted models accounted for 37 % of this geographical variation, with diabetes mortality higher in the Northwest (RR 2.55, 95 % CI 1.74, 3.73) and Northeast (RR 2.68, 95 % CI 1.70, 4.21) compared with the South. Diabetes mortality was higher in urbanized areas (RR tertile 3 versus tertile 1 (‘RRt3vs1’) 1.39, 95 % CI 1.17, 1.66), with higher mean body mass index (RRt3vs1 1.46, 95 % CI 1.18, 1.80) and with higher average temperatures (RR 1.05 95 % CI 1.03, 1.08). Diabetes mortality was lower where consumption of alcohol was excessive (RRt3vs1 0.84, 95 % CI 0.72, 0.99). No association was observed with smoking, overconsumption of red meat, high mean sedentary time, systolic blood pressure, cholesterol, and diabetes prevalence.

**Conclusions:**

Declines in diabetes mortality between 2006 and 2012 have been unequally distributed across China, which may imply differentials in diagnosis, management, and the provision of services that warrant further investigation.

**Electronic supplementary material:**

The online version of this article (doi:10.1186/s12889-015-1982-0) contains supplementary material, which is available to authorized users.

## Background

Recent studies have indicated substantial geographic variations and changes in the prevalence of diabetes in China over time, representing a key public health challenge [1–5]. In 2002 National Nutrition Survey, the prevalence of diabetes in adults was only 2.7 % [6], and in 2010 Chronic Disease Risk Factor Surveillance in China, the prevalence had increased to 9.7 % according to the same criteria [7]. This equates to approximately 100 million adult diabetes cases totally [3, 4]. Compared to the prevalence, the mortality attributable to diabetes is much lower, but has increased by 30 % from 8.0/100000 in 1990 to 11.2/100000 in 2010 based on the current Global Burden of Disease (GBD) estimates [8].

Concurrent to the accelerating rate of urbanization in the past two decades, China has (and continues to) experience epidemiological transition [9–11]. The burden of disease attributable to individual behaviors is steadily rising and inequitably [8, 12]. Unhealthy diets characterized by high intake of sodium and low intakes of fruit, vegetables and whole grains have synergized with decreasing levels of participation in physical activity [13–15]. These changes in built environment and behavior have been implicated in the observed increases in obesity and diabetes [2, 5, 8, 9].

There has been comparatively less research on trends in diabetes mortality, however, despite geographical inequity in the aforementioned risk factors likely to be driving local clusters of the diabetes epidemic. Understandings of where this avoidable form of mortality clusters would be highly valuable from the perspective of resource allocation for targeted prevention efforts. National mortality surveillance data showed that diabetes mortality was higher in urban than in rural areas, was highest in eastern region, and was lowest in western region [16]. It is not clear, though, whether the urban–rural disparity has widened, narrowed or remained stable over time, nor whether it is consistent in magnitude across all regions.

This report aims to address this gap in the literature by examining trajectories in diabetes mortality in China from 2006 to 2012 and by determining potential drivers for potential geographic variations. The questions addressed in this study were:

i) To what extent does diabetes mortality vary geographically across China, and has that variation changed overtime? (i.e. spatiotemporal variation)
ii) How much of the geographic variation in diabetes mortality is attributable to urbanization, person-level risk factors and environmental factors?

## Methods

### Study design

To address these research questions, an analysis of time trends and geospatial variation in diabetes mortality was implemented using nationally-representative mortality surveillance data. Diabetes mortality counts attributable to geographical areas were analyzed using multilevel models for assessment of the presence of spatiotemporal variation. The degree to which these spatiotemporal variations and urban/rural and regional inequalities were attributable to diabetes risk factors was assessed using a selection of area-level explanatory variables derived from the 2010 census and the 2010 China Chronic Disease Risk Factor Surveillance (CDRFS).

### Data and measures

The key outcome, type II diabetes mellitus (T2DM) mortality, was defined by the underlying cause of death using the International Classification of Disease 10 (ICD-10) range E10–E14. Mortality counts were obtained from the China Disease Surveillance Point System. The Disease Surveillance Point (DSP) system comprises 161 counties (or districts) across all 31 provinces, municipalities and autonomous regions in mainland China, providing coverage of approximately 73 million people. Previous work has demonstrated the representativeness of the DSP system [17]. Multiple strategies for addressing variation in data quality have been employed. Under-reporting and misclassification of cause of death are common challenges in sample mortality registration approaches, which have been addressed in two under-reporting field surveys conducted during 2006–2008 and 2009–2011 [18].

T2DM mortality counts were cross-classified by 5 year age group (>20y), gender, year (2006–2012 inclusive) and DSP. Corresponding population counts cross-classified by 5 year age group (>20y), gender and DSP were obtained from the Chinese census in 2010. China Census 2010 was used as a reference population for calculating age-standardized diabetes mortality rates. To account for population change across the study period, census data from 2000 to 2010 was used to calculate the annual rate of change in population for each of the 161 DSPs. The total population for each DSP in the years 2006–2012 inclusive was then estimated assuming an exponential growth across the time period.

Each of the 161 DSPs in the China Mortality Surveillance System were classified as ‘rural’ (*n* = 97) or ‘urban’ (*n* = 64) according to administrative characteristics. In addition, all DSP points were allocated to a regional classification by the China National Bureau of Statistics as follows: (i) South – Guangdong, Guangxi, Hainan; (ii) North - Beijing, Tianjin, Hebei, Shanxi, Inner Mongolia; (iii) East - Shanghai, Shandong, Jiangsu, Anhui, Jiangxi, Zhejiang, Fujian; (iv) Central - Hubei, Hunan, Henan; (v) Southwest - Chongqing, Sichuan, Guizhou, Yunnan, Tibet; (vi) Northwest - Shaanxi, Gansu, Ningxia, Xinjiang, Qinghai; and (vii) Northeast - Heilongjiang, Jilin, Liaoning.

Socioeconomic, environmental and behavioral risk factors for diabetes derived from the literature included behavioral risk factors such as smoking, alcohol use, sedentary lifestyles, diets, metabolic risk factors such as overweight/obesity, hypertension, cholesterol level, T2DM prevalence, and dyslipidaemia [19]. The urbanization rate [20] and average temperature [21] were also additional variables included in the analysis. The percentage of socioeconomic variables and risk factors were classified in 3 tertiles in order to examine for potentially curvilinear associations with diabetes mortality. Data from the CDRFS in 2010 [7] was used to estimate the prevalence of smoking, the prevalence of heavy alcohol consumption, sedentary time, the prevalence of red meat intake, the prevalence of DM, the mean SBP, the mean BMI and the mean cholesterol level of the adult population in each DSP. Diabetes was defined according to the ADA 2010 criteria as (i) a self-reported previous diagnosis by health professionals, (ii) fasting plasma glucose ≧ 126 mg/dL, (iii) 2-hour glucose ≧ 200 mg/dL, or (iv) HbA1c concentration of 6.5 % or more.(4) Cholesterol level was measured from blood samples collected from every participant after an overnight fast of over 10 h.

The urbanization rate of each DSP was from the national census in 2010. Several studies indicated that the temperature was highly correlated with the diabetes mortality [22–24], the higher of the temperature, the higher of the diabetes mortality. In our study, the average temperature of each DSP in 2010 was calculated with the routine monitoring data from the China Meteorological Administration.

### Ethics

The ethics committee of the Chinese Center for Disease Control and Prevention approved the 2010 CDRFS and written informed consent was obtained from each participant before data collection.

### Statistical analysis

Age-standardized diabetes mortality rates for each year across the study period were estimated overall, separately by gender, and between urban and rural DSPs. The average diabetes mortality from 2006 to 2012 of each region was calculated and presented visually, with slope estimates from regression models used to display the change in diabetes mortality over 2006–2012. Socioeconomic, environmental and behavioral factors were investigated in a series of multivariate negative binomial regression models, with cross-classified diabetes mortality counts (by age, gender, DSP and year) fitted as the outcome variable. Negative binomial regression was selected to account for over-dispersion of the mortality counts. The natural logarithm of the equivalently classified denominator counts were fitted as an offset to account for local population distributions.

As the analysis involved repeated observations of the same DSPs across seven consecutive years, random intercepts were applied to adjust regression parameters and 95 % confidence intervals (95 % CIs) for temporally auto-correlated residuals [25]. These ‘multilevel models’ also facilitated the estimation of geographic variation between DSP points was expressed in the form of a median rate ratio (MRR) [26]. An MRR can be interpreted in the same way as a rate ratio (RR); MRRs equal to 1 suggest no geographic variation in the outcome variable, whereas values above 1 indicate the necessity of taking context into account.

Trends in diabetes mortality were investigated using a model including fixed effects adjustment for age, gender and year. Potential curvilinear trajectories through time were investigated using quadratic transformations of the year variable. The regional differences in trends were investigated initially by fitting a random slope for the year variable on each DSP. Collective interpretation of the intercept variance, slope variance, covariance and associated standard errors indicated the extent to which diabetes mortality varied spatiotemporally. Further exploration involved adjusting for fixed effects of the region variables to investigate temporal variations in diabetes mortality by context.

To investigate the role of socioeconomic, behavioral, metabolic risk factors and environmental factors associated with diabetes mortality, the urbanization rate, behavioral risk factor prevalence (smoking, alcohol consumption, sedentary time, red meat intake), metabolic factors (prevalence of T2DM, mean SBP, mean BMI, mean cholesterol level) and the average temperature for each DSP were added sequentially to regression models. Contributions of each of these variables towards explaining variations in diabetes mortality were recorded using the proportional change in the intercept (i.e. DSP) variance. All fixed effect parameters were exponentiated to RRs and 95 % CIs. All statistical analyses were conducted in MLwIN v2.30.

## Results

**Fig. 1:**
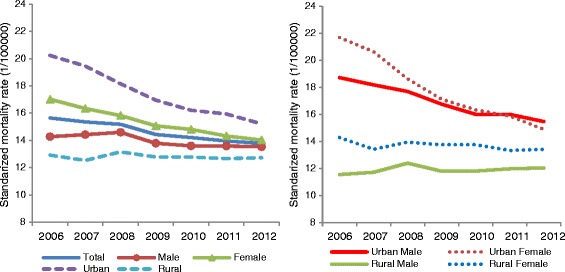
Diabetes mortality trends during 2006–2012 in male/female and urban/rural

Age-standardized diabetes mortality rates among people aged 20 years and older decreased 12 % (from 15.65 to 13.78 per 100,000) from 2006 to 2012 (Table 1). Gender differences were also observed, with diabetes mortality decreasing more slowly for males (0.8 per 100,000) than females (3.0 per 100,000). The disparity between urban and rural residence was notable. Diabetes mortality during the study period decreased by 2 % (from 12.92 to 12.72) in rural areas, but decreased by 25 % (from 20.24 to 15.21) in urban areas. Gender and urban/rural stratified analyses showed the mortality was consistently lowest in rural males during the study period, followed by rural females (Figure 1). The mortality of urban females decreased sharply compared to urban males, although the mortality of urban females was higher than urban males in 2006(21.7 and 18.73 per 100000 respectively), and the mortality of urban females was lower than that of urban male at the end of the period.

**Table 1:** Age-standardized mortality rate per 100,000 of diabetes from 2006 to 2012 in men/women and urban/rural

Year	Urban			Rural			Total		
	Male	Female	Total	Male	Female	Total	Male	Female	Total
2006	18.73	21.70	20.24	11.55	14.28	12.92	14.27	17.02	15.65
2007	18.19	20.65	19.45	11.73	13.42	12.53	14.42	16.34	15.35
2008	17.70	18.63	18.15	12.40	13.95	13.15	14.59	15.83	15.18
2009	16.76	17.12	16.95	11.80	13.76	12.78	13.79	15.07	14.43
2010	16.01	16.34	16.21	11.81	13.76	12.77	13.60	14.81	14.21
2011	16.00	15.88	15.94	11.98	13.34	12.66	13.58	14.33	13.95
2012	15.47	14.90	15.21	12.04	13.42	12.72	13.53	14.02	13.78

**Fig. 2:**
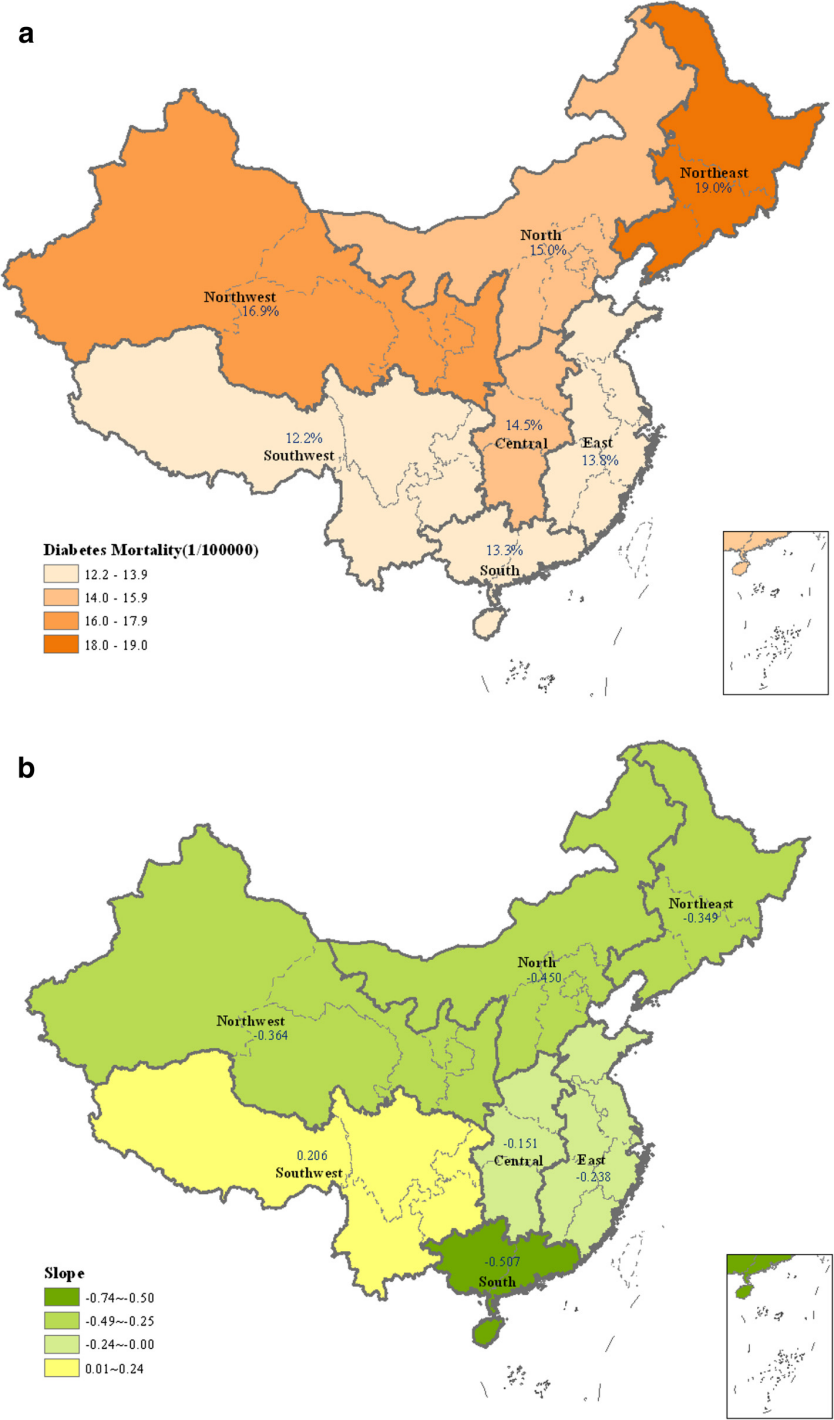
**a** Distribution of Diabetes Mortality among 7 Regions in China. **b** Geographic distribution of diabetes mortality changes in China from 2006 to 2012

As shown in Figure 2a, North–south differences were observed on the age-standardized diabetes mortality. The top 3 regions of age-standardized diabetes mortality were Northeast, Northwest and North respectively (Table 2), the lowest is southwest.

**Table 2:** Age-standardized mortality rate per 100,000 of diabetes from 2006 to 2012 in 7 regions classified by urban and rural areas

	North		East		Central		South		Southwest		Northwest		Northeast	
	Urban	Rural	Urban	Rural	Urban	Rural	Urban	Rural	Urban	Rural	Urban	Rural	Urban	Rural
2006	20.1	14.1	20.1	12.2	23.0	12.6	25.8	9.7	10.5	10.8	23.0	15.3	20.0	18.7
2007	22.7	9.5	17.6	12.3	19.3	12.5	21.5	10.7	12.8	10.1	25.3	16.5	22.3	18.7
2008	19.4	11.9	15.4	12.7	20.2	12.4	20.3	9.4	15.4	13.0	22.7	16.3	21.7	19.0
2009	16.3	11.1	14.5	12.2	23.9	12.4	17.8	10.4	13.3	11.1	18.0	16.2	20.9	20.3
2010	16.0	11.6	14.7	12.7	20.1	12.8	17.2	10.4	10.5	12.4	18.7	15.1	19.6	15.1
2011	18.6	10.7	14.2	12.5	20.2	12.4	17.4	9.9	11.2	12.9	15.2	13.4	17.3	17.5
2012	14.8	10.3	13.7	12.8	16.5	11.9	15.8	11.1	13.6	13.0	18.3	15.9	18.8	16.5
Total	18.0	11.2	15.4	12.5	20.3	12.4	19.2	10.3	12.6	12.0	19.8	15.5	20.0	17.9

As shown in Figure 2b, the diabetes mortality decreased in nearly all the regions except southwest, but the trend had statistical significance only in south, north, east, northeast and northwest. The decreasing slope was highest in the south (0.74, 95 % CI 0.43 ~ 1.04) and the north (0.66, 95 % CI 0.35 ~ 0.96). Though there was an upward slope (0.29, 95 % CI −0.09 ~ 0.68) in the southwest, the trend was not statistically significant. Details can be found in the Additional file 1: Table S1.

The multilevel model #1 (Table 3) indicates that age- and gender-adjusted diabetes mortality decreased between 2006 and 2012 (RR0.98, 95 % CI 0.97, 0.99). Diabetes mortality was higher among women and increased with age. The DSP intercept variance was statistically significant and important (MRR = 1.47). The slope variance was small but the intercept/slope covariance was non-trivial (0.268, SE 0.007), indicating diverging trajectories in diabetes mortality manifesting at the DSP scale. Approximately 9 % of the DSP-level variation in diabetes mortality was explained by adjustment for region in model 2 and the MRR was 1.45. Diabetes mortality was significantly higher in the Northwest (RR 1.45, 95 % CI 1.10, 1.90) and Northeast (RR 1.42, 95 % CI 1.07, 1.87) compared with the south. To explore the role of socioeconomic factors on the diabetes mortality, urbanization rate of each DSP was added. Comparing with the low urbanization rate areas, the RR and 95 % CI of the areas with moderate and high urbanization rate were 1.09(0.93, 1.27) and 1.41(1.21, 1.64) respectively (model 3). The prevalence of lifestyle factors accounted for 27 % of the variation (model 4).

**Table 3:** Spatiotemporal variation in diabetes mortality in China during 2006–2012

	Model1 (time, age, gender)	Model2 (adding region, region*time)	Model3 (adding urbanization rate)	Model4 (adding behavior risk factors)	Model5 (adding metabolic risk factors)	Model6 (adding average temperature)
Fixed effects						
Time(years)	0.98(0.97,0.99) *	0.98(0.97,0.99) *	0.98(0.97,1.00) *	0.98(0.97,1.00) *	0.98(0.97,1.00) *	0.98(0.97,1.00) *
Age (5 year groups)	1.64(1.64,1.65) *	1.64(1.64,1.65) *	1.64(1.63,1.65) *	1.64(1.63,1.65) *	1.64(1.63,1.65) *	1.64(1.63,1.65) *
Gender (ref: male)	1	1	1	1	1	1
female	1.06(1.03,1.08) *	1.05(1.03,1.08) *	1.06(1.03,1.08) *	1.06(1.03,1.08) *	1.06(1.03,1.08) *	1.05(1.03,1.08) *
Region(ref:south)		1		1	1	1
North		1.06(0.81,1.38)	1.11(0.86,1.43)	1.06(0.82,1.38)	0.96(0.70,1.31)	1.49(1.02,2.16) *
East		1.12(0.88,1.43)	1.14(0.90,1.46)	1.09(0.85,1.39)	1.10(0.85,1.41)	1.40(1.06,1.84) *
Central		1.24(0.95,1.62)	1.33(1.03,1.73) *	1.27(0.97,1.65)	1.22(0.93,1.59)	1.49(1.12,1.97) *
Southwest		0.95(0.73,1.23)	1.07(0.83,1.38)	1.15(0.90,1.48)	1.14(0.88,1.47)	1.43(1.08,1.88) *
Northwest		1.45(1.10,1.90) *	1.61(1.24,2.10) *	1.54(1.17,2.01) *	1.48(1.11,1.97) *	2.55(1.74,3.73) *
Northeast		1.42(1.07,1.87) *	1.51(1.16,1.97) *	1.50(1.13,1.99) *	1.39(1.00,1.92) *	2.68(1.70,4.21) *
Urbanization rate(ref:low)			1	1	1	1
Moderate			1.09(0.93,1.27)	1.14(0.98,1.33)	1.14(0.99,1.33)	1.11(0.97,1.29)
High			1.41(1.21,1.64) *	1.50(1.26,1.79) *	1.42(1.18,1.71) *	1.39(1.17,1.66) *
% smokers (ref: low)				1	1	1
Moderate				0.90(0.77,1.04)	0.94(0.80,1.11)	0.95(0.81,1.11)
High				0.90(0.77,1.06)	1.00(0.84,1.19)	1.02(0.86,1.21)
% over drinkers(ref:low)				1	1	1
Moderate				0.97(0.83,1.13)	0.93(0.80,1.08)	0.89(0.77,1.03)
High				0.87(0.74,1.03)	0.85(0.72,1.00)	0.84(0.72,0.99) *
% eat red meat(ref:low)				1	1	1
Moderate				0.91(0.79,1.06)	0.92(0.79,1.07)	0.91(0.79,1.06)
High				0.86(0.72,1.03)	0.88(0.74,1.06)	0.91(0.76,1.08)
Sedentary time(ref:low)				1	1	1
Moderate				1.06(0.92,1.22)	1.06(0.92,1.22)	1.05(0.92,1.20)
High				1.09(0.91,1.29)	1.04(0.87,1.24)	1.01(0.85,1.20)
Body mass index (ref: low)					1	1
Moderate					1.13(0.96,1.33)	1.22(1.04,1.43) *
High					1.23(1.00,1.51) *	1.46(1.18,1.80) *
SBP level(ref:low)					1	1
Moderate					0.93(0.79,1.10)	0.93(0.80,1.09)
High					0.95(0.78,1.15)	1.01(0.84,1.21)
Cholesterol level(ref:low)					1	1
Moderate					0.99(0.86,1.14)	0.99(0.86,1.13)
High					1.00(0.86,1.15)	0.97(0.84,1.12)
DM Prevalence(ref:low)					1	1
Moderate					1.08(0.92,1.27)	1.01(0.86,1.18)
High					1.07(0.89,1.29)	0.99(0.83,1.18)
Average temperature						1.05(1.03,1.08) *
Random effects						
DSP intercept variance (standard error)	0.166(0.019) *	0.151(0.018) *	0.137(0.016) *	0.121(0.014) *	0.113(0.014) *	0.104(0.013) *
MRR	1.47	1.45	1.42	1.39	1.38	1.36
PCV		9 %	17 %	27 %	32 %	37 %
DSP slope variance by year (standard error)	0.006(0.001) *	0.007(0.001) *	0.006(0.001) *	0.006(0.001) *	0.006(0.001) *	0.006(0.001) *
Intercept/Slope Covariance	0.268 (0.007)	0.274 (0.007)	0.279 (0.007)	0.269 (0.007)	0.268 (0.007)	0.257(0.007)

* *p* < 0.05, *MRR* Median Rate Ratio, *PCV* proportional change in variance (PCV) in model x compared to model 1

DSP: counties (rural) and districts (urban)

In models incorporating the four metabolic risk factors (average BMI, systolic pressure, Cholesterol level and the DM prevalence), only average BMI was positively associated with diabetes mortality (tertile 2 RR 1.13, 95 % CI 0.96, 1.33, tertile 3 RR 1.23 95 % CI 1.00, 1.51)(model 5). The average temperature was positively associated with the diabetes mortality (RR 1.05, 95 % CI 1.03, 1.08). Adjustment of the average temperature amplified the magnitude of effect size of regional differences and BMI. All the fitted risk factors accounted for 37 % of the DSP-level variation. These adjustments had negligible explanatory power for the DSP slope variance and the intercept/slope covariance.

## Discussion

Based on nationally representative surveillance sample, we found diabetes mortality was higher in female than in male, urban areas than rural areas. Diabetes mortality decreased over the period 2006–2012 in females and in urban areas but did not change substantially for males and in rural areas. There was also substantial geographic variation in diabetes mortality across China’s major geographic regions. North–south differences were notable for age-standardized diabetes mortality, with northeast and northwest areas having higher diabetes mortality rates compared to the southern areas after adjustment for potential health-related factors. Higher mortality rates of diabetes were associated with DSP-level urbanization rate, average BMI level and average temperature.

The prevalence of diabetes has been shown to be higher in urban than rural areas [3, 4] and findings from the present study suggest a similar pattern for diabetes mortality. In China, most cause-specific mortality has been shown to be higher in rural than in urban areas, and in males than in females [27]. The pattern of diabetes mortality, however, differs substantially from this general mortality pattern. Previous smaller scale, provincial studies have also reported similar patterns in diabetes mortality. For example, in Henan [28], Sichuan [29], Guangxi [30] and Hunan province [31], diabetes mortality was higher in urban than in rural areas, and among females than males. The present study is the first study to investigate geographic differences in diabetes mortality across the whole country.

We found the mortality increased with the higher degree of urbanization. With the rapid development of Chinese economy, great changes have taken place in people’s diet structure, especially the urban population. They eat more meat and milk with high quantity of calories, higher fat and protein, less whole grains, vegetables. The similar change of dietary pattern has been seen in other emerging economies, such as the BRICS (Brazil, the Russian Federation, India, China and South Africa) countries [32–34]. These lifestyle changes provide the possibility of increasing prevalence of diabetes [3, 4, 9]. The beneficial effect of lifestyle modification with calorie restriction shown in diabetes prevention program was reported in both Chinese and Indian population [35, 36].

Although diabetes mortality was higher in urban areas, declines were observed between 2006 and 2012 and converged with rates in rural areas. This trend is not consistent with trends in risk factors over this period, such as BMI, hypertension, diabetes prevalence and lipid level. The study in Henan showed similar results [31]. The declining mortality trend in urban areas is likely to reflect improvements in health infrastructure and institutional care as well as palliative and hospice care services. Recent data in the general population show that there is a trend towards decreased cardiovascular events and increased life expectancy. This also applies for people living with type 2 diabetes. However, increased survival in the general population is associated with epidemics of obesity and sedentary lifestyle all over the globe, leading to a higher incidence of type 2 diabetes worldwide. This counteracts the diminution of diabetes-related mortality that would move forward on an ascending slope in the next decades [37].

The higher mortality in the north in comparison with the south of China was partly due to the difference of diet culture between these two regions. Compared with the south, people in north had higher body mass index, sodium intake, sodium/potassium ratio, and higher intake of calcium, magnesium, phosphorus, and vitamins A and C [38, 39].

We found a positive association between BMI and diabetes mortality, which is also consistent with results from individual-level prospective studies [40, 41] and the positive association between temperature and diabetes mortality was also reported in developed countries [21, 42]. In addition, the positive association between the urbanization rate and diabetes mortality was also reported in other study [20]. Urbanization has led to changes in patterns of human activity, diet, and social structures in China, with profound implications for non-communicable diseases—eg, diabetes, cardiovascular disease, cancer, and neuropsychiatric disorders. He and colleagues’ study [43] in a geographically and socially isolated ethnic minority group in southwest China provided early evidence of the effect of urbanization on chronic disease; age-related increases in blood pressure were greater in individuals who had moved to urban areas than in those who remained in rural villages [43]. Since then, researchers have suggested that Chinese urban environments promote lifestyles that place people at risk of hypertension, obesity and diabetes—risk factors for many non-communicable diseases.

A key strength of this study is the nationally representative data on diabetes counts across 161 DSPs. The mortality data was consistent and reliable after deletion of DSPs with poor data quality and adjustment of under-reporting. To our knowledge, this is the first study investigating the spatiotemporal variation of diabetes mortality in a nationally representative data in China. The use of multilevel model was another important strength, as it not only accounted for the correlated nature of the time-series analysis through adjusting for repeated measurement of DSPs between 2006 and 2012, but it also afforded the disentangling of contextual associations from those attributable to compositional factors.

There are limitations in this study. Using mortality counts based on the underlying cause of death might underestimate the true mortality from diabetes, because people with diabetes often develop complications and experience multi-morbidities. To ensure the accuracy of the underlying death causes coding, we provided routine training to coding staff and had a regular checking and verification process to avoid coding errors. Secondly, this is an ecological study of a mortality time series and the risk factor adjustment was limited to population-level point estimates derived from the CDRFS and the 2010 census. Change in diabetes mortality coinciding with change in these population-level exposures therefore could not be assessed.

## Conclusions

In conclusion, this study reports spatiotemporal variation in diabetes mortality between 2006 and 2012. The findings from this study suggest that diabetes mortality remained higher in the north than the south across the study period. The regional disparities in diabetes mortality and risk factors in China reaffirm the heterogeneous composition of the population and the uneven distribution of biological and social determinants of diabetes mortality in the country. These wide variations of diabetes mortality and risk factors underscore the need to develop different strategies for different geographic areas of the country to manage this important cause of disability and death in China.

## Additional file

Additional file 1: Table S1. Age-standardized mortality in different regions of China 2006–2012.
