# Supplementary material for: Spatiotemporal variation in diabetes mortality in China: multilevel evidence from 2006 and 2012
Source: BMC Public Health. 2015 Jul 10;15:633. doi: 10.1186/s12889-015-1982-0 (PMC4496807; doi:10.1186/s12889-015-1982-0)
Supplement: Additional file 1: Table S1. — Age-standardized mortality in different regions of China 2006–2012. [file 12889_2015_1982_MOESM1_ESM.docx]

Appendix table 1: Age-standardized mortality in different regions of China 2006-2012.

| **Year** | **North** | **East** | **Central** | **South** | **Southwest** | **Northwest** | **Northeast** |
| --- | --- | --- | --- | --- | --- | --- | --- |
| 2006 | 17.1 | 15.3 | 15.5 | 16.6 | 10.7 | 17.8 | 19.5 |
| 2007 | 16.9 | 14.7 | 14.4 | 15.0 | 10.9 | 19.0 | 20.6 |
| 2008 | 16.1 | 14.0 | 14.3 | 13.1 | 13.8 | 18.3 | 20.4 |
| 2009 | 13.9 | 13.2 | 15.2 | 13.1 | 11.9 | 16.8 | 20.6 |
| 2010 | 14.1 | 13.6 | 14.9 | 13.2 | 11.7 | 16.4 | 17.5 |
| 2011 | 15.0 | 13.2 | 14.5 | 11.4 | 12.3 | 14.0 | 17.5 |
| 2012 | 12.9 | 13.2 | 13.2 | 12.1 | 13.2 | 16.8 | 17.8 |
| Total | 15.0 | 13.8 | 14.5 | 13.3 | 12.2 | 16.9 | 19.0 |
| Slope | -0.66 | -0.35 | -0.22 | -0.74 | 0.29 | -0.53 | -0.51 |
| Low CL | -0.96 | -0.50 | -0.45 | -1.04 | -0.09 | -1.00 | -0.91 |
| Upper CL | -0.35 | -0.20 | 0.02 | -0.43 | 0.68 | -0.07 | -0.10 |
